# The critical role of tumor microbiome in cancer immunotherapy

**DOI:** 10.1080/15384047.2024.2301801

**Published:** 2024-01-19

**Authors:** Liu Yang, Qi Wang, Lijuan He, Xingyu Sun

**Affiliations:** a School of Clinical Medicine, The Affiliated Hospital, Southwest Medical University, Luzhou, China; b Department of Gastroenterology, Affiliated Hospital of Jiangsu University, Jiangsu University, Zhenjiang, China; c Department of Health Management Center, The Affiliated Hospital, Southwest Medical University, Luzhou, China; d Department of Gynecology, The Affiliated Traditional Chinese Medicine Hospital, Southwest Medical University, Luzhou, China

**Keywords:** Biomarkers, cancer immunotherapy, immune modulation, Tumor microbiome, microbial-based therapies, interdisciplinary research

## Abstract

In recent years, the microbiome has shown an integral role in cancer immunotherapy and has become a prominent and widely studied topic. A full understanding of the interactions between the tumor microbiome and various immunotherapies offers opportunities for immunotherapy of cancer. This review scrutinizes the composition of the tumor microbiome, the mechanism of microbial immune regulation, the influence of tumor microorganisms on tumor metastasis, and the interaction between tumor microorganisms and immunotherapy. In addition, this review also summarizes the challenges and opportunities of immunotherapy through tumor microbes, as well as the prospects and directions for future related research. In conclusion, the potential of microbial immunotherapy to enhance treatment outcomes for cancer patients should not be underestimated. Through this review, it is hoped that more research on tumor microbial immunotherapy will be done to better solve the treatment problems of cancer patients.

## Introduction

Cancer immunotherapy has recently become a groundbreaking method for treating malignancies, it aims to use the host’s immune system to identify and eliminate tumor cells, significantly different from traditional cancer therapies such as radiotherapy and chemotherapy, which directly target cancer cells.^1^ The concept of cancer immunotherapy has its roots in the late 19th century, marked by William Coley’s observation of cancer regression in patients with postoperative bacterial infections.^2^ Over the decades, this foundational understanding evolved, leading to the development of advanced immunotherapies in recent years, including immune checkpoint inhibitors, chimeric antigen receptor (CAR) T-cell therapy, cancer vaccination, and oncolytic virus therapy.^3^ In the past few decades, cancer immunotherapy has shown remarkable effects in the treatment of melanoma, non-small cell lung cancer renal cell carcinoma, and Hodgkin’s lymphoma.^4^ Of particular note, the advent of immune checkpoint inhibitors targeting programmed cell death protein 1 (PD-1) and cytotoxic T-lymphocyte-associated protein 4 (CTLA-4) has brought about a major shift in the treatment of advanced melanoma, greatly improving survival rates.^5^ Despite the remarkable success of immunotherapy in cancer treatment, not all patients benefit from it, and some may even suffer serious immune-related adverse events.^6^ Therefore, a comprehensive understanding of the factors that influence the effectiveness and safety of tumor immunotherapy is critical, and in recent years, there has been an increasing number of studies on the impact of tumor microbiome on host immune response.^7^ To comprehensively address the role of microbiomes in cancer immunotherapy, this review distinguishes between the tumor microbiome and the gut microbiome. While both are integral to understanding cancer’s interaction with the host’s microbial environment, their distinct roles necessitate separate consideration. The tumor microbiome, directly situated within the tumor microenvironment, interacts uniquely with cancer cells and immune responses, in contrast to the more indirect influence of the gut microbiome.^8^

The tumor microbiome refers to the collective community of microorganisms, comprising bacteria, fungi, viruses, and protozoa, that reside in the tumor microenvironment.^9^ Accumulating evidence suggests that these microorganisms possess the capacity to impact the progression of the tumor and the host’s immune response directly or indirectly, thereby significantly affecting the efficacy and safety of cancer immunotherapy.^10,11^ For example, specific bacteria in the gut microbiome can enhance the therapeutic effect of immune checkpoint inhibitors by stimulating the activation and infiltration of cytotoxic T cells,^12^ and patients with higher gut microbiome diversity and abundance showed better clinical outcomes in cancer immunotherapy, highlighting the potential for microbiome regulation to improve immunotherapy efficacy.^13^ Thus, a deeper understanding of the complex interplay between the tumor microbiome, host immune response, and cancer immunotherapy is critical to facilitate the development of more effective and customized treatment options.

**Figure 1.**
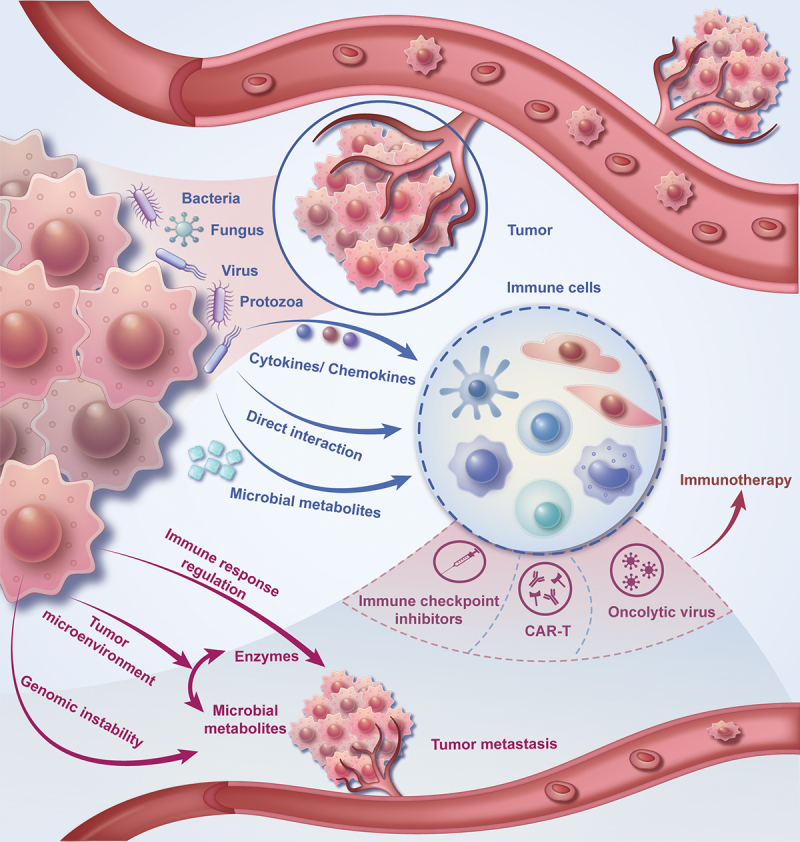
The composition of the tumor microenvironment, the mechanism of microbial immune regulation, the influence of tumor microorganisms on tumor metastasis, and the interaction between tumor microorganisms and immunotherapy.

**Figure 2.**
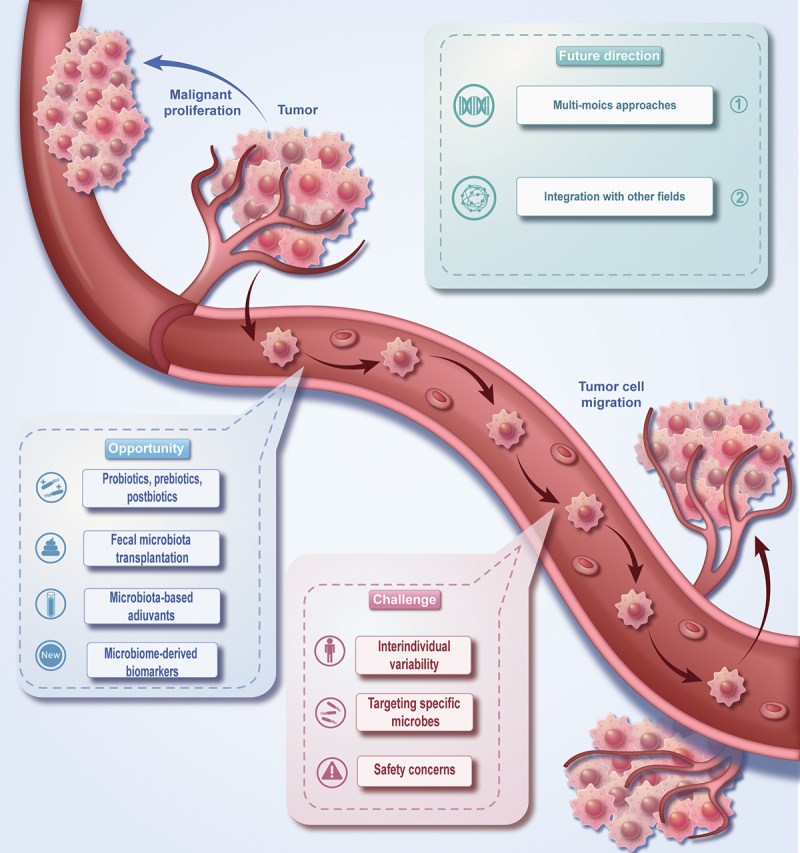
The challenges and opportunities of tumor microbiome in immunotherapy, and the prospect of future research.

This review summarized the composition of the tumor microenvironment, the mechanism of microbial immune regulation, the influence of tumor microorganisms on tumor metastasis, and the interaction between tumor microorganisms and immunotherapy (Figure 1). Besides, this review also emphasizes the challenges in our current comprehension of the tumor microbiome and its role in cancer immunotherapy, accentuating the areas that offer opportunities for future exploration and introducing the direction and prospect of future related research (Figure 2).

## The role of tumor microbiome in immune response regulation and tumor progression

### Composition and diversity of the tumor microbiome

The tumor microbiome refers to the collective community of microorganisms. The alterations in the immunological response of the host and the progression of the neoplasm can be a result of the interplay between these microorganisms and the tumor cells, as well as the surrounding microenvironment.^9^ The composition and diversity of the tumor microbiome varies among different types of cancer and can be influenced by the location of the tumor, host genetics, immune status, and environment.^14^ A gram-negative anaerobic bacterium, Fusobacterium nucleatum, is highly enriched in the tumor tissue of colorectal cancer patients and is associated with a poor prognosis.^15^ Similarly, the microbiota content in breast tumors differed significantly from the microbiota content in adjacent normal tissues and found a large increase in gram-positive bacteria (i.e. staphylococcus and streptococcus).^16^ Consequently, the tumor microbiome exhibits considerable variability in its composition. High-throughput sequencing technologies, such as 16S rRNA gene sequencing and metagenomic sequencing, can help judge the expression information of cells in the microbiome of various cancer tumors and reveal the relationship of cell populations in the microenvironment.^17^ A detailed understanding of the complex interactions between the tumor microbiome and the immune system is crucial for developing innovative therapeutic strategies to improve the effectiveness of cancer immunotherapy.

Recent advances highlight the significance of the tumor-associated microbiome in directly shaping anticancer immunity and influencing checkpoint immunotherapy outcomes. Unlike the gut microbiome, the tumor microbiome directly interacts with cancer cells and immune cells, forming a unique ecological niche that significantly impacts the tumor’s behavior and response to therapies. This article delves into how local tumor microbiota contributes to these processes, emphasizing the need to focus on the tumor-associated microbiome in addition to the gut compartment.^8^

### Mechanisms of microbiome-mediated immune modulation

The microbiome residing in tumors can modulate the immune response through various mechanisms, including but not limited to direct interaction with immune cells, the production of metabolites, and the induction of cytokines and chemokines.^12^ This suggests a complex and multifaceted role of the tumor microbiome in shaping immune responses.

Direct interaction between microbes and immune cells has a significant influence on the activation, differentiation, and functionality of various immune cell types, such as T cells, B cells, dendritic cells, and macrophages.^18^ Specific bacterial species can induce the activation of dendritic cells, resulting in the production of pro-inflammatory cytokines and subsequent activation of T cells. This can promote an anti-tumor immune response by upregulating the cytotoxic activity of CD8+ T cells, as well as the secretion of interferon-gamma (IFN-γ).^19^

Microbial metabolites, such as short-chain fatty acids (SCFAs), also play a role in modulating the immune response. SCFAs are produced by the fermentation of dietary fiber by gut bacteria and have been shown to not only inhibit the activation of nuclear factor kappa B (NF-κB) and facilitate the differentiation of regulatory T cells (Tregs) but also to enhance the performance of immune checkpoint blockade therapies, particularly in the context of dietary fiber intake.^20,21^ These metabolites can modulate the expression of immune checkpoint molecules, including programmed cell death protein 1 (PD-1) and its ligand PD-L1, which are crucial targets in cancer immunotherapy.^22^

Besides direct interactions and metabolite production, the tumor microbiome can also impact the immune response by stimulating the release of various cytokines and chemokines. Specifically, certain strains of bacteria possess the capability to stimulate the interleukin-6 (IL-6), interleukin-10 (IL-10), and transforming growth factor-beta (TGF-β), consequently augmenting tumor proliferation and evading immune surveillance through targeted inhibition of cytotoxic T cells and natural killer (NK) cells.^23^

Recent studies have highlighted the significant impact of diet on the gut microbiome, which subsequently influences the immune system. Dietary interventions, particularly those involving high-fiber and fermented foods, have been shown to modulate the composition and function of the gut microbiota. For example, a high-fiber diet can increase the abundance of microbiome-encoded glycan-degrading carbohydrate-active enzymes (CAZymes), indicating a shift toward a microbiota capable of breaking down complex polysaccharides. Interestingly, while such a diet maintains microbial community diversity, it can lead to different immunological responses based on the baseline microbiota diversity of the individual. On the other hand, a diet rich in fermented foods has been observed to enhance microbiota diversity consistently and reduce markers of inflammation. This suggests that fermented foods could play a crucial role in reversing the trends of reduced microbiome diversity and heightened inflammation commonly seen in industrialized societies.^24^ These findings underscore the potential of dietary strategies in shaping the gut microbiome and, by extension, modulating immune responses, which could have implications for cancer immunotherapy.

Beyond the gut microbiome, the tumor-associated microbiome emerges as a critical player in modulating local immune responses. This compartmentalized microbiome directly influences the tumor microenvironment, affecting cytokine and chemokine production, and consequently, the immune landscape. Understanding these interactions is crucial for developing targeted therapies to manipulate the tumor microbiome, potentially enhancing the efficacy of immunotherapies.^8^

In general, the tumor microbiome can modulate the immune response via a variety of mechanisms, acquiring a thorough understanding of these intricate mechanisms will facilitate the development of therapeutic strategies that harness the tumor microbiota for cancer immunotherapy.

### Effects of tumor microbiome on cancer progression and metastasis

Several mechanisms, including but not limited to the regulation of local and systemic immune responses, alterations in the tumor microenvironment, and the induction of genomic instability, can promote or hinder the progression and metastasis of cancer.^25^ This underscores the multifaceted nature of cancer development and the complexity of its interactions within the body.

The regulation of immune response can influence tumor progression by altering the balance between pro-inflammatory and anti-inflammatory cytokines.^10^ For example, certain bacterial species possess the capacity to stimulate the production of pro-inflammatory cytokines, such as interleukin-17 (IL-17), which can participate in both tumor development and metastasis, through the recruitment of tumor-associated macrophages and neutrophils.^26^

The tumor microbiome can influence cancer progression through multiple mechanisms. These include the production of enzymes by microorganisms that degrade the extracellular matrix, facilitating tumor invasion and metastasis,^11^ and the generation of microbial metabolites affecting pH, oxygenation status, and nutrient accessibility, impacting cell proliferation, survival, and migration.^27^ Moreover, other complex interactions involving immune modulation, alteration of gene expression within cancer cells, and the competitive exclusion of beneficial microbiota are also critical in this process.

Additionally, cancer progression and metastasis might be further accelerated by the tumor microbiome via the induction of genomic instability. Bacterial genotoxins, such as Escherichia coli’s colibactin, can cause DNA damage and genomic instability in the host cells, thereby leading to the accumulation of mutations and the development of carcinogenesis.^28^

The tumor microbiome exerts a consequential impact on the progression and metastasis of cancer through various pathways. A comprehensive understanding of these mechanisms presents promising prospects for novel therapeutics targeting the tumor microbiome in cancer therapy.

## Interaction between the tumor microbiome and immunotherapies

### Impact of the microbiome on the efficacy of immune checkpoint inhibitors

Immune checkpoint inhibitors (ICIs) are a class of cancer immunotherapies that work by blocking inhibitory pathways, such as the programmed cell death protein 1 (PD-1) and cytotoxic T-lymphocyte-associated protein 4 (CTLA-4) pathways, fostering anti-tumoral immune responses.^29^ Although the exact mechanism by which the microbiome affects the efficacy of ICI has not yet been elucidated, many previous studies have shown that specific bacterial species can effectively alter the immune response, particularly by promoting the recruitment and activation of immune cells such as dendritic cells, CD8+ T cells, and natural killer cells, thereby enhancing the anti-tumor effects of ICIs.^12,22^ Two recent studies have highlighted the microbiome can affect the efficacy of ICIs, and found that specific microbial species are associated with desirable therapeutic outcomes. In melanoma patients subjected to anti-PD-1 therapy, a conspicuous increase in the abundance of particular bacterial species, namely Faecalibacterium and Ruminococcaceae, was correlated with better treatment response and longer progression-free survival.^7^ Similarly, a higher abundance of Akkermansia muciniphila was associated with appreciably improved clinical outcomes in non-small cell lung cancer and renal cell carcinoma patients subjected to anti-PD-1 therapy.^30^ This strongly suggests that the microbiome plays a substantial role in regulating the efficacy of ICIs and that some specific bacterial species are associated with enhanced response to treatment. More comprehensive studies are needed to explore the potential of manipulating the microbiome to enhance the efficacy of cancer immunotherapy.

### Effects of the microbiome modulation on CAR-T cell and other immune cell immunotherapies

The role of the tumor microbiome in modulating the efficacy of CAR-T cell therapy and other cellular immunotherapies is an emerging area of research. Chimeric antigen receptor T (CAR-T) cell therapy is a form of cellular immunotherapy that involves engineering patient-derived T cells ex vivo to express CARs, which target specific tumor-associated antigens.^31^ Remarkable success has been demonstrated by CAR-T cell therapy in the treatment of hematological malignancies, including B-cell acute lymphoblastic leukemia (B-ALL) and diffuse large B-cell lymphoma (DLBCL).^32^ Additionally, other cellular immunotherapy approaches like tumor-infiltrating lymphocyte (TIL) therapy and natural killer (NK) cell therapy are also being investigated for their potential to treat solid tumors.^33^

Notably, recent studies have suggested that specific gut microbiota can exert a significant influence on the activation, expansion, and function of T cells and NK cells.^34,35^ In light of these findings, it is increasingly apparent that the microbiome may play a critical role in shaping the immune response and, consequently, influencing the efficacy of cellular immunotherapy. For instance, preclinical studies have demonstrated that the presence of specific commensal bacteria can enhance the efficacy of adoptive T-cell therapy in mouse models of cancer.^22^ Similarly, another study elucidated the potential for the gut microbiota to modulate the anti-tumor effects of NK cell-based immunotherapy in a mouse model of melanoma.^36^

Despite the encouraging discoveries, the exact mechanisms through which the tumor microbiome influences the efficacy of CAR-T cell therapy and other cellular immunotherapies remain obscure. Further research is needed to elucidate these mechanisms and determine whether microbiome modulation could be harnessed to improve the efficacy of cellular immunotherapies in cancer treatment.

### Microbial influences on cancer vaccines and oncolytic virus therapy

Cancer vaccines aim to activate the immune system to recognize and target cancer cells by identifying specific tumor-associated antigens. Prophylactic vaccines, primarily used to prevent cancer-causing infections such as HPV, are designed to stop the development of cancer. In contrast, therapeutic vaccines are developed to combat existing cancers by targeting neoantigen-specific or tumor-associated antigens.^37^ Oncolytic virus therapy, on the other hand, involves the use of genetically modified or naturally occurring viruses that selectively infect and kill cancer cells while sparing healthy cells.^38^

Emerging evidence indicates that the tumor microbiome could potentially have a significant impact on the effectiveness of cancer vaccines and oncolytic virus therapy. Research has demonstrated that the existence of particular bacterial species in the gut has a direct influence on the activation and function of dendritic cells, which have a vital role in initiating immune responses and antigen presentation.^34^ Consequently, there is an indication that the microbiome could regulate the efficiency of cancer vaccines by modifying the capability of the immune system to recognize and respond to tumor antigens. Furthermore, various studies have revealed that certain bacterial species are capable of directly interacting with oncolytic viruses, thereby affecting their replication and spread in the tumor microenvironment.^39^ In a preclinical study, a mouse model of melanoma displayed that the presence of specific commensal bacteria could enhance the therapeutic efficacy of an oncolytic virus.^40^

While these findings highlight the potential role of the microbiome in influencing the efficacy of cancer vaccines and oncolytic virus therapy, further research is needed to understand the fundamental mechanisms and explore potential clinical applications. Discovering particular microbial signatures that enhance or hinder these immunotherapies could result in the innovation of strategies to enhance their efficacy in cancer management.

## The challenge of tumor microbiome in immunotherapy

### Interindividual variability and the need for personalized approaches

One of the primary challenges to using the tumor microbiome for immunotherapy is the high degree of interindividual variability in the microbiome composition across different patients.^10^ This variability can be attributed to several factors, including but not limited to age, diet, lifestyle, genetics, and the use of antibiotics, which can collectively impact the composition and function of an individual’s microbiome.^41^ The specific microbial profile may confer benefit to one patient, but not necessarily be advantageous for another patient owing to differences in their immune responses and microbiome compositions. This highlights the need to develop individualized approaches when designing microbiome-based cancer therapies.^42^

Personalized approaches encompass the identification of specific microbial signatures closely linked with favorable treatment outcomes in individual patients. This information can be used to develop tailored treatments, such as fecal microbiota transplantation (FMT) or the use of specific probiotics, to alter the patient’s microbiome and improve the effectiveness of immunotherapies.^43,44^ Nevertheless, the development of individualized strategies requires a more comprehensive understanding of the complex interactions between the host, tumor, microbiome, and immune system. To overcome the challenges related to interindividual variability, there is a pressing need for large-scale multi-omic studies that converge genomic, transcriptomic, proteomic, and metabolomic information obtained from diverse patient cohorts. Such studies can help elucidate the functional significance of specific microbial taxa and their metabolites in modulating the immune response and treatment outcomes, ultimately enabling the development of more personalized and effective microbiome-based cancer therapies.

In addition to personalized approaches, it is important to recognize the potential of broader strategies that may benefit a wide range of patients. For instance, therapies aimed at increasing overall bacterial diversity in the microbiome, though not tailored to individual microbial profiles, have shown promise in enhancing the effectiveness of immunotherapies. Such approaches can help mitigate the challenges posed by interindividual variability by employing general improvements in microbial diversity, which may in turn enhance immune system responsiveness. This suggests a dual strategy in microbiome-based cancer therapies: personalization where specific microbial profiles are known and more general approaches to improve microbial diversity for a wider patient population.

### Identifying and targeting specific microbial species or communities

Another challenge encountered in utilizing the tumor microbiome for immunotherapy is the identification and targeting of specific microbial species or communities that can regulate the immune response and the cancer progression. The vast complexity of the microbiome, which comprises numerous bacterial, fungal, and viral species, makes it difficult to pinpoint the exact microbial taxa that may be driving a particular response.^10^ The present methodologies for discerning relevant microbial species or communities rely on high-throughput sequencing technologies, such as 16S rRNA gene sequencing and metagenomic shotgun sequencing, which provide insights into the taxonomic composition and functional potential of the microbiome.^45^ Nonetheless, these methods may have certain limitations, including biases in amplification, difficulties in precisely assigning taxonomy, and an inability to deduce active microbial functions or the actual metabolic condition of the microbiome.^46^ To overcome these limitations, researchers are integrating multi-omics methodologies, including meta-transcriptomics, meta-proteomics, and metabolomics, to achieve deeper comprehension concerning the functional interactions existing between the microbiome and host immune system.^47^ Additionally, in-vitro and in-vivo experimental models, such as germ-free mice or humanized mouse models, can help elucidate the causal role of specific microbial taxa in modulating immune responses.^11^

Targeting individual microbial species or communities for therapeutic purposes can be a significant challenge, owing to the complex ecological interactions that exist within the microbiome. Strategies to manipulate the microbiome, such as the use of probiotics, prebiotics, antibiotics, or fecal microbiota transplantation, may have unintended consequences on the overall microbial community and host health.^48^ It is therefore imperative to gain a deeper understanding of the ecological principles that govern the microbiome, as well as develop interventions that are more precise and targeted, to harness the tumor microbiome for cancer immunotherapy in an efficacious manner.

### Overcoming potential off-target effects and safety concerns

Potential off-target effects and safety concerns are also among the challenges of using tumor microbes for cancer immunotherapy. Given that the tumor microbiome is profoundly interdependent with the host’s immune system and overall health, interventions targeting specific microbial species or communities could potentially trigger unintended consequences.^10^ For instance, the use of broad-spectrum antibiotics to modulate the microbiome may engender dysbiosis, a state of microbial imbalance that has been linked with various diseases and health complications.^34^ Furthermore, the administration of live bacterial strains as probiotics may carry the risk of bacteremia or infection, particularly in individuals with compromised immune systems.^49^

To surmount these challenges, researchers are concentrating on formulating more accurate and targeted approaches to modulate the tumor microbiome. For instance, narrow-spectrum antibiotics or bacteriophages can be used to selectively target specific bacterial species without causing significant disruptions to the overall microbial community.^50^ Additionally, engineered bacteria or oncolytic viruses could be designed with built-in safety mechanisms, such as inducible suicide genes or gene circuits that limit their proliferation.^51^ Subsequent research ought to concentrate on formulating precise, targeted, and safe strategies to handle the microbiome.

## The opportunities of tumor microbiome in immunotherapy

### Probiotics, prebiotics, and postbiotics

Probiotics, prebiotics, and postbiotics are some of the new prospects for regulating the tumor microbiota and promoting the efficacy of immunotherapy. Probiotics are living microorganisms that, when administered in sufficient quantities, bestow a positive health impact on the host.^52^ They have been shown to enhance gut health, boost the immune system, and potentially modulate the tumor microenvironment.^53^ Numerous studies have illustrated the possibility of probiotics in improving the reaction to cancer immunotherapies, including immune checkpoint inhibitors.^22^

Prebiotics refer to the indigestible components found in food that selectively stimulate the growth or activity of beneficial bacteria in the gut.^54^ They can be utilized to manipulate the composition of the tumor microbiome, promoting the growth of bacterial species that enhance the effectiveness of cancer immunotherapies.^7^

Postbiotics are non-viable microbial products or by-products that exert beneficial effects on the host’s health,^55^ such as bacterial cellular components, extracellular vesicles, or metabolites produced during the fermentation process. Postbiotics have demonstrated potential in modulating the immune response and enhancing the efficacy of cancer immunotherapies.^53^

While these show great potential for modulating the tumor microenvironment and increasing the effectiveness of cancer treatment, further scientific exploration is needed to elucidate the specific mechanisms by which these interventions trigger their effects. Furthermore, the formulation of personalized approaches, which take into account the microbiome’s interindividual variability, will be crucial for maximizing the therapeutic potential of these strategies.

### Fecal microbiota transplantation

Fecal microbiota transplantation (FMT) is a procedure that involves transferring fecal material containing healthy gut microbiota from a donor to a recipient, with the aim of reestablishing a balanced gut microbial community. FMT has evinced striking efficacy in combating recurrent Clostridium difficile infections, and its prospective use in the field of oncology treatment has garnered increasing attention.^56^ Recent studies suggest that FMT can enhance the efficacy of cancer immunotherapies, most notably immune checkpoint inhibitors, by modulating the gut microbiome and, consequently, the tumor immune microenvironment.^10,43^ In preclinical models, FMT derived from cancer patients exhibiting positive responses to immune checkpoint inhibitors instilled into germ-free mice showed augmented antitumor immunity and improved therapeutic outcomes.^57^ Early-phase clinical trials have also exhibited promising findings, as some patients demonstrated improved immunotherapy responses after receiving FMT from responsive donors.^58^

Besides, FMT has been identified as a potential adjunctive therapy to other cancer treatments, such as chemotherapy and radiotherapy, by mitigating their side effects and enhancing their efficacy through microbiome modulation.^59^ Nevertheless, the clinical application of FMT for cancer treatment encounters multiple challenges about the need for standardized protocols, donor selection criteria, and long-term safety assessments. Moreover, the optimal timing, frequency, and route of administration for FMT in cancer patients necessitate further study.^60^ Despite these challenges, FMT is an emerging and promising strategy for harnessing the tumor microbiome to improve cancer treatment outcomes.

### Microbial-based adjuvants

Microbiota-based adjuvants have lately emerged as a prospective strategy to augment the effectiveness of existing immunotherapies. By harnessing the immunomodulatory traits of specific microbial constituents or entire microbes, these adjuvants have the potential to enhance the immune response against cancer cells and improve clinical outcomes. Some bacterial-derived substances, such as CpG oligodeoxynucleotides and flagellin, have manifested their ability to stimulate the host immune system and consequently, improve the efficiency of cancer vaccines and immune checkpoint inhibitors.^61^ These microbial components can activate the pattern recognition receptors (PRRs) on immune cells, leading to the initiation of downstream signaling pathways and the induction of pro-inflammatory cytokines.^62^ This heightened immune response can, in turn, enhance the recognition and destruction of cancer cells.

Whole microbes, such as attenuated strains of bacteria or viruses, can also serve as adjuvants to improve the efficacy of immunotherapies. For example, the use of oncolytic viruses that have been modified to express immune-stimulatory molecules has demonstrated favorable outcomes in preclinical studies and early-phase clinical trials.^63^ These oncolytic viruses can directly infect and eliminate cancer cells while simultaneously activating the immune system via the secretion of tumor antigens and pro-inflammatory cytokines.^64^

Moreover, probiotics, including live biotherapeutic products (LBPs) that consist of live, nonpathogenic bacteria, are being explored as adjuvants to enhance the efficacy of existing immunotherapies. These probiotics and LBPs offer potential benefits in modulating the immune response to improve the outcomes of cancer treatments. Preclinical studies have demonstrated the potential of certain LBPs to modulate the tumor microenvironment and increase the infiltration of immune cells, ultimately leading to improved responses to immunotherapies.^22^

Overall, microbial-based adjuvants present a positive approach to bolster the efficacy of existing immunotherapies. Subsequent research is necessary to identify the most effective components or strains, optimize their delivery, and determine the best combination strategies with current immunotherapies to maximize the therapeutic outcomes.

### Microbiome-derived biomarkers

The identification and development of microbiome-derived biomarkers for predicting treatment response is a burgeoning area of research with significant potential for individualizing cancer therapy. By characterizing a subject’s tumor microbiome, it may be possible to predict their response to specific immunotherapies and accordingly tailor treatment strategies. Several studies have reported the correlation between particular microbial taxa and the reaction to immunotherapy. For example, patients with a higher abundance of certain commensal bacteria, such as Akkermansia muciniphila, have been found to show more favorable reactions to immune checkpoint inhibitors.^30^ Likewise, the existence of specific Bifidobacterium species has been linked to enhanced responses to anti-PD-L1 therapy in preclinical models.^22^ Beyond individual microbial taxa, specific microbial signatures or functional profiles have the potential to serve as potential biomarkers. With the advances in metagenomic sequencing and bioinformatics, the characterization of the functional potential of the microbiome can be thoroughly facilitated, enabling the identification of specific metabolic pathways or gene clusters correlated with treatment response.^65^

Furthermore, the development of microbiome-derived biomarkers is not limited solely to the tumor microbiome. The gut microbiome has also been linked to the modulation of the effectiveness of cancer treatments, and specific gut microbial signatures have been associated with the response to immunotherapies in both preclinical and clinical studies.^7^ Despite these encouraging discoveries, additional research is necessary to validate and refine these potential biomarkers in more extensive and varied patient cohorts. Moreover, the integration of microbiome-derived biomarkers with other clinical, genomic, and immunologic factors will be imperative in generating more precise and comprehensive predictive models.

## Future perspectives and research directions

### Advancements in high-throughput sequencing and multi-omics approaches for characterizing the tumor microbiome

The future advancement in comprehending the tumor microbiome and its function in cancer immunotherapy heavily depends on the enhancements in high-throughput sequencing and multi-omics approaches. These approaches facilitate a comprehensive and in-depth description of the complex microbial communities and their interactions with the host immune system.

Next-generation sequencing (NGS) technologies have revolutionized our comprehension of the microbiome, providing high-resolution taxonomic and functional profiles of microbial communities.^66^ Innovations in NGS techniques, including single-cell sequencing and long-read sequencing, hold great potential in augmenting the resolution and accuracy of microbiome analyses, enabling the identification of novel microbial species and strain-level differences which could hold substantial implications for the therapeutic results in cancer treatment.^67^ In addition to genomics, other omics approaches, such as metatranscriptomics, metaproteomics, and metabolomics, provide valuable insights into the functional activities of microbial communities within the tumor microenvironment. The integration of these datasets can disclose the complex mechanisms between microbial metabolism, host immune response, and cancer progression.^47^

Machine learning and bioinformatics tools are essential in analyzing and interpreting the vast amount of multi-omics data generated from these approaches. Sophisticated computational algorithms can discern patterns and correlations between the tumor microbiome and treatment response, while simultaneously uncovering potential mechanistic connections and causative associations.^68^ Furthermore, the integration of multi-omics data with clinical and immunologic information can expedite the creation of predictive models and biomarkers, thereby ultimately enabling personalized cancer treatment strategies.^42^

In summary, progressions in high-throughput sequencing and multi-omics approaches are critical for advancing our understanding of the tumor microbiome and its significance in cancer immunotherapy. Continued investment in these technologies, and their integration with other research disciplines, will be essential for unlocking the full potential of microbiome-based therapies in cancer treatment.

### Integrating microbiome research with other emerging fields in cancer therapy

As our comprehension of the tumor microbiome continues to broaden, it is becoming increasingly clear that integrating microbiome research with other emerging fields in cancer therapy is essential to maximize the potential of these innovative methodologies. This integration will not only expedite the development of more effective and personalized interventions, but also provide a more profound insight into the intricate interplay between microbial communities, the tumor microenvironment, and the host immune response.

One burgeoning field that harbors immense potential for synergy with microbiome research is cancer genomics, particularly in the context of tumor neoantigen mimicry. The genetic and epigenetic modifications in tumor cells create a unique molecular fingerprint, which can be exploited for therapeutic targets.^69^ Additionally, microbial species within the gut and beyond can produce epitopes resembling tumor neoantigens, influencing the immune response and the outcomes of immune checkpoint blockade.^70,71^ The integration of cancer genomics and microbiome data, therefore, can reveal critical insights into how microbiome-mediated tumor neoantigen mimicry may affect checkpoint immunotherapy outcomes, opening new avenues for precision medicine in cancer treatment.

Cancer immunology, another rapidly advancing domain, explores the pivotal role of the immune system in cancer development, progression, and response to treatment. It has been demonstrated that the tumor microbiome exerts influence on the immune response of the host, a deeper understanding of these intricate interactions may foster the development of more effective immunotherapies, including immune checkpoint inhibitors, chimeric antigen receptor (CAR)-T cell therapy, and cancer vaccines.^71^

The promising field of nanotherapeutics, entailing the application of nanoparticles for drug delivery and imaging, also benefits from merging with microbiome research. For example, nanoparticle-based drug delivery systems can be meticulously engineered to specifically target microbial components within the tumor microenvironment, thus potentially enhancing the efficacy of cancer treatments while minimizing side effects.^72^

The integration of microbiome research with systems biology methodologies can offer a holistic comprehension of the complex interactions between the tumor microbiome, host immune response, and cancer progression. Systems biology leverages computational and mathematical models to study complex biological systems and holds immense potential in predicting treatment responses and identifying potential biomarkers for precision medicine.^73^

In summary, the integration of microbiome research with other emerging fields holds great promise for the development of innovative and personalized cancer treatments. Continued interdisciplinary research and collaboration will be crucial to fully harness the potential of these novel approaches.

## Conclusion

In this review, we have underscored the pivotal significance of the tumor microbiome in modulating the immune response and its impact on cancer treatment. Harnessing the tumor microbiome offers significant potential for improving cancer treatment outcomes. As our understanding of the intricate interplay between the tumor microbiome, immune system, and cancer cells continues to deepen, novel therapeutic approaches can be devised to target these interactions and enhance the efficacy of existing treatments.

As we advance in the field of cancer immunotherapy, the distinction between tumor and gut microbiomes becomes increasingly relevant. Future research should explore the unique pathways through which the tumor microbiome affects immunotherapy, aiming to develop strategies for therapeutic modulation of these microbial communities within the tumor microenvironment. A deeper understanding of the tumor microbiome’s composition and function will refine our approach to cancer treatment, moving closer to precision medicine for solid tumors.
